# Atrial Fibrillation Post Cardiac Surgery Trends Toward Management

**DOI:** 10.4103/1995-705X.73212

**Published:** 2010

**Authors:** Awad A. R. Alqahtani

**Affiliations:** Department of Cardiology and Cardiothoracic Surgery, Hamad Medical Corporation, Doha, Qatar

**Keywords:** Atrial fibrillation, cardiac surgery, antiarrhythmia

## Abstract

Post operative atrial fibrillation (POAF) is more common than before due to increased numbers of cardiac surgeries. This in turn is associated with increased incidence of post operative complication, length of hospital stay and subsequent increase the cost of hospitalization. Therefore preventing and/or minimizing atrial fibrillation by pharmacological or nonpharmacological means is a reasonable goal. POAF has also been associated with postoperative delirium and neurocognitive decline. The precise pathophysiology of POAF is unknown, however most of the evidence suggests it is multifactorial. Different risk factors have been reported, and many studies have evaluated the prophylactic effects of different interventions. This review article highlights the incidence, risk factors, and pathogenesis, prevention, and treatment strategies of POAF.

## INTRODUCTION

Postoperative Atrial Fibrillation (POAF) is more common than before due to the increased number of cardiac surgeries. This in turn is associated with an increased incidence of postoperative complications, length of hospital stay, and subsequent increase in the cost of hospitalization. Therefore, preventing and / or minimizing atrial fibrillation by pharmacological or non-pharmacological means is a reasonable goal.[1,2]

Postoperative atrial fibrillation has also been associated with postoperative delirium and neurocognitive decline.[3,4] The precise pathophysiology of POAF is unknown, however, most of the evidence suggests that it is multifactorial. Different risk factors have been reported, and many studies have evaluated the prophylactic effects of different interventions. This review article highlights the incidence, risk factors, pathogenesis, prevention, and treatment strategies of POAF.

## THE EXTENT OF THE PROBLEM

The true incidence of postoperative atrial fibrillation (POAF) following cardiac surgery is unclear. Reported incidences range from 10 - 65%. This range is wide, because studies that examined Atrial Fibrillation (AF) following coronary artery bypass graft (CABG) differ in baseline patient characteristics, type of surgery, methods of detection, and definitions of AF.[5] Overall, it is estimated that the incidence of POAF is approximately 30% after pure CABG surgery, 40% following valve replacements or repair, and increases to approximately 50% after combined CABG / valvular procedures. It is expected that the incidence will rise in the future, as the population going for cardiac surgery is getting older and the incidence of AF in general is age-dependent. POAF tends to occur within two to four days after the procedure, with the peak incidence on the second postoperative day. Of the patients who experienced an arrhythmia, 70% developed it before the end of the fourth postoperative day and 94% before the end of the sixth postoperative day.[6] Furthermore, POAF was estimated to prolong hospital stay by almost 4.9 days, and hence, the cost of POAF on hospital resources was significant.[6]

## THE RISK FACTORS FOR POAF

**Figure 1:**
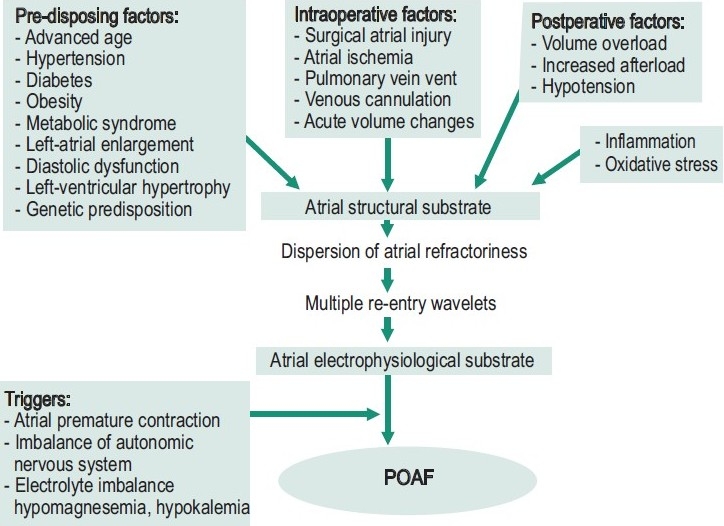
POAF pathogenesis

Numerous studies have identified risk factors associated with the development of atrial fibrillation following cardiac surgery.[7] Risk factors such as older age, previous history of AF, male gender, decreased left-ventricular ejection fraction, valvular heart surgery, left-atrial enlargement, chronic obstructive pulmonary disease, chronic renal failure, diabetes mellitus, and rheumatic heart disease are associated with development of atrial fibrillation [Figure 1].[1,3,7–9]

## THE MECHANISMS OF POAF

Atrial fibrillation is attributed to enhanced automaticity in one or several rapidly depolarizing foci and re-entry involving one or more circuits.[10] Its development is likely to be multifactorial and has not yet been fully identified. A proposed mechanism like pericardial inflammation, autonomic imbalance during the postoperative period, excessive production of catecholamines, and a fluid shift with resultant changes in volume and pressure, are all attributed to the development of POAF [Figure 1].[11–18]

Some studies indicated that patients with postoperative AF could have pre-existing electrophysiological disturbances.[19,20] Mariscalco *et al*,[21] suggested a link between atrial histopathology before the surgical procedure and postoperative AF. The abnormalities found include: cytoplasmatic vacuolization, interstitial fibrosis, and nuclear derangement of myocytes. These findings supported the hypothesis that postoperative atial fibrillation occurred in the presence of AF vulnerability (triggers) and the ability to maintain AF (substrate) was associated with pre-existing degenerative changes. Moreover, multiple re-entry wavelets resulting from the dispersion of atrial refractoriness seemed to be the electrophysiological mechanism of POAF. Another mechanism such as neurohormonal activation increased the susceptibility to POAF.[4] Increased sympathetic and parasympathetic activation altered atrial refractoriness (e.g., shortening of the atrial effective refractory period), possibly contributing to the arrhythmia substrate.[16]

Some studies suggest that patients with RR interval variability are at risk of developing POAF. These findings suggest that interventions that alter both the sympathetic and parasympathetic nervous systems may be beneficial in suppressing this postoperative arrhythmia. In addition, there are supporting studies revealing that underlying inflammation plays an important factor in the pathogenesis of POAF.[11,12] Supporting this hypothesis is the finding that extracorporeal circulation contains systemic inflammatory mediators that may be responsible for the development of POAF. Some studies have reported that an elevated white cell count, which is usually seen in the days following cardiopulmonary bypass, is an independent predictor for the occurrence of POAF.[13,14] Other studies have revealed that early occurrence of POAF is linked to an increased inflammatory response, post cardiac surgery.[22–24] Inflammation, inhomogeneity of atrial conduction, and incidence of POAF are significantly decreased by anti-inflammatory treatment with prednisone.[11] Surgical trauma to the atria is associated with an increased incidence of POAF, which explains why patients undergoing valvular surgery have the highest risk of developing POAF.[1,8] Some studies suggest that less manipulation of the atria decreases atrial inflammation, and subsequently, AF.[25]

## DO WE NEED TO TREAT POAF?

Considering that POAF is associated with a higher incidence of heart failure, stroke, prolonged hospital stay, and increased costs, it is justifiable to treat it. In a retrospective study, the Texas Heart Institute of Cardiovascular Research database was used to identify patients who developed POAF. AF was diagnosed in 16% (n = 994) of the population (n = 6475) and was associated with greater in-hospital mortality, more strokes, and prolonged hospital stays.

## MODALITIES FOR PREVENTION OF POAF

Several studies have evaluated the effectiveness of pharmacological and non-pharmacological interventions to prevent or decrease the high incidence of POAF. In 2006, the American College of Cardiology, the American Heart Association (AHA), and the European Society of Cardiology [Table 1].[26,27] jointly published a guideline for the prevention and management of POAF.

**Table 1:** Indications for intervention in AF post cardiac surgery according to the ACC/AHA/ESC guidelines

Indication Class I	Unless contraindicated, tretment with an oral beta-blocker drug to prevent POAF is recommeded for patients undergoing cardiac surgery.	Level of Evidence: A
	Administration of AV nodal blocking agents is recommended to archieve rate control in patients who develop POAF.	Level of Evidence: B
Indication Class IIa	Preoperative administration of amiodarone reduces the incidence of AF in patients undergoing cardiac surgery and represents appropriate prophylactic therapy for patients at high risk for POAF.	Level of Evidence: A
	It is reasonable to restore sinus rhythm by phramacologic cardioversion with ibutilide or direct- current cardioversion in patients who develop POAF, as advised for nonsurgical patients.	Level of Evidence: B
	It is resonable to administer antiarrhythmic medications in an attempt to maintain sinus rhythm in patients with recurrent of refractory POAF, as recommended for other patients who develop AF.	Level of Evidence: B
	It is reasonable to administer antithrombotic medication in patients who develop POAF, as recommended for nonsurgical patients.	Level of Evidence: B
Indication Class IIb	Prophylactic administration of sotalol may be considered for patients at risk of developing AF after cardiac surgery.	Level of Evidence: B

ACC = American College of Cardiology; AHA = American Heart Association; ESC = European Society of Cardiology; AV = atrio ventricular.

### Beta-blockers

Beta-blockers have been the most studied drugs to date, for the prevention of POAF. These drugs are primary in AF prevention and should be used routinely in every patient.[28,29] Several clinical trials have evaluated the effect of various beta-blockers on the incidence of POAF,[30–32] and the results indicate an overall reduction of this complication. However, it should be noted that even in recent large trials where this strategy has been widely applied, the incidence of POAF remains nearly 60% in selected patients. This in turn supports the need for further preventive strategies in addition to beta-blockade. Sotalol is a beta-blocker that has important class III antiarrhythmic effects and has also been effective in the prevention of POAF, both when compared with placebo[33] and with other beta blockers, to elucidate the specific class III action.[34,35] However, the side effects of sotalol, such as hypotension and bradycardia, and in particular, its proarrhythmic effects, has limited its use in perioperative management.

### Amiodarone

Amiodarone is a class III antiarrhythimic drug that also has beta and alpha adrenergic-blocking properties. It plays a role in controlling the sympathetic overstimulation seen in patients undergoing cardiac surgery. A short perioperative course of oral amiodarone added to a routine beta-blockade has proved to be a very promising approach of management. This combination therapy was associated with a 50% lower incidence of postoperative atrial tachyarrhythmia in patients undergoing valve replacement / repair and /or CABG. In a large prospective trial, the Prophylactic Oral Amiodarone for the Prevention of Arrhythmias that Begin Early After Revascularization, Valve Replacement, or Repair (PAPABEAR), the number needed to treat was only 7.5, to prevent one patient from developing POAF.[36] The result of the PAPABEAR trial were consistent with the pooled results of a meta-analysis of 19 trials comparing amiodarone with placebo.[37] In these 19 trials, AF was reduced by 50% in the amiodarone group (95% confidence interval [CI], 0.43 to 0.59; *P* < 0.0001); Furthermore, there was also a significant reduction in ventricular tachyarrhythmia, strokes, and hospital stay. The authors concluded that in the absence of a contraindication, prophylactic amiodarone should be implemented as a routine therapy for high-risk patients undergoing cardiac surgery.[37]

### Atrial pacing

There are three mechanisms by which atrial pacing decreases and / or prevents AF:

- Avoiding the trigger for AF by suppressing atrial premature beats
- Reduction of bradycardia-induced dispersion of atrial depolarization that contributes to the electrophysiological substrate for AF
- Using dual atrial pacing, such mechanisms minimize and / or prevent the development of intra- atrial entry, and hence, AF. Meta-analyses,[38–40] demonstrated a promising outcome on using single- or dual-site atrial pacing, which reduces the risk of new-onset POAF. However, the number of patients participating in these studies was small, and the protocol used for the pacing sites varied widely among these studies.

## OTHER MEDICATIONS

### Calcium-channel blockers

Numerous studies have evaluated the nondihydropyridine calcium-channel blocker. The finding shows that calcium-channel blockers reduce the risk of supraventricular tachyarrhythmia. However, some studies that suggested using this drug preoperatively found an increased incidence of atrioventricular (AV) block and low-output syndrome, which was attributed to the negative inotropic and chronotropic effects of this medication. Therefore, the use of these agents should be considered with caution until more information on their safety profile become available.

### Statins

Several studies point out that statins reduce inflammation in patients with coronary artery disease. Observational studies have previously suggested that patients on statin therapy have a lower incidence of POAF after CABG.[41] The prospective, randomized study, Atorvastatin for Reduction of MYocardial Dysrhythmia After cardiac surgery (ARMYDA-3)[42] demonstrated that treatment with atorvastatin (40 mg/day), started seven days before elective cardiac surgery under cardiopulmonary bypass and continued in the postoperative period, reduced the occurrence of POAF by 61%.

### N-3 polyunsaturated fatty acids

Experimental studies in rats and dogs showed that polyunsaturated fatty acids (PUFAs) have significant antiarrhythmic effects on the atrial muscle.[43,44] Furthermore, in a 12-year follow-up study from the general population; consumption of fish that induces high plasma concentrations of PUFA has been associated with a lower incidence of AF.[45]

A randomized trial by Calo *et al*,[46] showed that in 160 patients who underwent elective CABG, PUFA supplementation significantly lowered the incidence of POAF. The effect was similar to that seen when using beta-blockers, sotalol or amiodarone.

### Anti-inflammatory agents

Corticosteroids have anti-inflammatory properties; hence it has been studied for the treatment of POAF. Clinical trials in the past have shown significant reduction in the incidence of POAF in those patients who received steroid compared to controls. There is an increase in inflammatory mediators postoperatively, which predisposes the development of POAF in susceptible patients. A multicenter trial,[47] consisting of 241 consecutive patients undergoing cardiac surgery, was randomized to receive either 100 mg hydrocortisone or placebo. The incidence of POAF during the first 84 hours was significantly lower in the hydrocortisone group (36 of 120; 30%) than in the placebo group (58 of 121; 48%), and the adjusted Hazard Ratio (HR) was 0.54 (95% CI, 0.35 to 0.83; P = 0.004).

### Magnesium

A meta-analysis by Miller *et al*,[48] suggested that giving a magnesium supplement was effective for reducing POAF. Its efficacy in reducing AF was similar to that obtained from common antiarrhythmic drugs. However, the small numbers of patients used in these studies, and the design variability of the studies, limited the interpretation of these study results and their clinical application.

### N-acetylcysteine

N-acetylcysteine (NAC) is an antioxidant agent that minimizes cellular oxidative damage.[49,50] It has also been shown that NAC may reduce reperfusion arrhythmias, ischemia / reperfusion injury,[51] and / or extension of infarction. The use of NAC with reperfusion therapy in patients with acute myocardial infarction has also been associated with less oxidative stress and better preservation of left ventricular function.[52] NAC has also shown beneficial effects in chronic pulmonary disease,[49] which is considered another risk factor for POAF. A study published in the *European Heart Journal* two years ago.[50] suggested the potential benefits of using NAC perioperatively and its continued infusion for 48 hours postoperatively, to reduce the incidence of POAF.

### TREATMENT OF POAF

Although POAF is transient and self-limiting, treatment is indicated for patients who are hemodynamically unstable, and who develop cardiac ischemia or heart failure. The current treatment includes control of ventricular rate, and restoring / maintaining sinus rhythm in addition to prevention of thromboembolism.

### Rhythm control

Most of the recent evidence suggests that rhythm control is better than rate control.

The reason being that rhythm control maintains the patients in sinus rhythm, with decreasedlength of hospital stay.[53] Different agents may beeffective in converting AF to sinus rhythm [Table 2], includingamiodarone,[54] procainamide,[55] ibutilide,[56] and sotalol.[57] In one study,[56] ibutilide was more effective thanplacebo for the treatment of POAF, but polymorphicventricular tachycardia was reported, which was attributed to electrolyteimbalance. In the postoperative period, the beta blockingaction of sotalol was particularly effective inreducing the ventricular rate and its proarrhythmic toxicity was relatively infrequent, but this agent seemed less effective than the others for inducing cardioversion of AF.

**Table 2:** Drugs used for rhythm control in atrial fibrillation

Drugs	Adult dosage	Advantages	Side effects
Amiodarone	2.5-5 mg/kg IV over 20 min then 15 mg/kg or 1.2 g over 24 h	Can be used in patients with severe LV dysfunction	Thyroid and hepatic dysfunction, torsades de pointes, pulmonary fibrosis, photosensitivity, bradycardia
Procainamide	10-15 mg/kg IV up to 50 mg/min	Therapeutic leavels quickly achieved	Hypotension, fever, accumulates in renal failure, can worsen heart failure, requires drug-level monitoring
Ibutilide	1 mg IV over 10 min, can repeat after 10 min ifno effect	Easy to use	Torsades de pointes more frequent than with amiodarone and procainamide

IV = intravenous; LV = left ventricular.

### Electrical cardioversion

If POAF results in acute heart failure, myocardial ischemia or hemodynamic instability urgent electrical cardioversion should be performed. It should also be considered electively to restore sinus rhythm after the first onset of AF, if the pharmacologic trial has failed to resume a sinus rhythm.

### Rate control

The postoperative recovery period is characterized by increased autonomic nervous system activity and adrenergic stress, and this in turn makes it difficult to control the ventricular rate in patients with POAF. Beta blockers are the therapy of choice, particularly in patients with ischemic heart disease; however, beta blockers are relatively contraindicated or poorly tolerated in patients known to have asthma or Bronchospasms, acute decompensated heart failure, or high-grade atrioventricular (AV) conduction block. Alternatively, other AV nodal blocking agents can be tried instead. Their dosages and side effects are presented in [Table 3].

**Table 3:** Drugs used for rate control in atrial fibrillation

Drugs	Adult dosage	Advantages	Side effects
Digoxin	0.25-1.0 mg IV then 0/125-0.5 mg/day IV or PO	Can be used in heart failure	Nausea, AV block moderate effect in POAF
Beta-blockers			
Esmolol	500 µg/kg over 5 min, then 0.05-0.2 mg/ kg/min	Short-acting effect and short duration	Might worsen congestive heart failure; cause bronchospasm, hypotension; AV block
Atenolol	1-5 mg IV over 5 min, repeat after 10 min then 50-100 mg bid PO	Rapid onset of rate control (IV)	
Metoprolol	1-5 mg IV over 2 min, then 50-100 mg bid PO	Rapid onset of rate control (IV)	
Calcium-channel blockers			
Verapamil	2.5-10 mg IV over 2 min, then 80-120 mg/day bid PO	Short-acting effect	Might worsen congestive heart failure, AV block
Diltiazem	0.25 mg/kg IV over 2 min, then 5-15 mg/h IV		

### Thromboembolism prevention

Postoperative Atrial Fibrillation is associated with an increased risk of perioperative strokes,[58,59] but this may be reduced by therapeutic anticoagulation. In contrast, anticoagulation in the postoperative period may increase the risk of bleeding or cardiac tamponade.[60] No controlled trials have specifically evaluated the efficacy and safety of anticoagulation therapy for new-onset POAF, which often resolves spontaneously after four to six weeks. Generally, anticoagulation is instituted for prolonged (> 48 hours) and / or frequent POAF episodes. The American College of Chest Physicians recommends the use of anticoagulation therapy, particularly for high-risk patients, such as those with a history of stroke or transient ischemic attacks, in whom AF develops after surgery. In these patients, it is also recommended to continue anticoagulation therapy for a further 30 days after return to normal sinus rhythm.[60]

## CONCLUSION

Postoperative Atrial Fibrillation is the most common arrhythmia after cardiac surgery. The frequency of this arrhythmia is increasing, most likely due to the increasing number of elderly patients undergoing cardiac surgery.

Currently, there are significant variations in the prevention strategies for POAF, with varied supportive evidence. The most recent evidence suggests that beta-blockers are effective, safe, and can be used in most patients. Therefore, unless contraindicated, beta-blockers should be continued perioperatively or initiated in all patients. In addition, amiodarone, statins, N-3 PUFAs, or NAC, may be used with beta-blocker as an adjunctive therapy. Such a combination therapy has been shown to be beneficial in reducing POAF.
